# Editorial: Respiratory diseases and management in livestock

**DOI:** 10.3389/fvets.2024.1367128

**Published:** 2024-01-19

**Authors:** Annamaria Pratelli, Francesco Cirone, Maria Mountricha, Barbara Padalino

**Affiliations:** ^1^Department of Veterinary Medicine, University of Bari Aldo Moro, Valenzano, Italy; ^2^Division of Animal Sciences, Department of Agricultural and Food Sciences, Alma Mater Studiorum, University of Bologna, Bologna, Italy

**Keywords:** livestock, respiratory diseases, welfare, management, prophylaxis

Among the challenges in the livestock industry, respiratory diseases can lead to significant economic losses and compromise animal health and welfare (1). These diseases can affect various species (cattle, pigs, horses, sheep, and goats), and their etiology may involve a range of agents such as viruses, bacteria and fungi (2, 3). Moreover, in cases of bacterial infections the use of antibiotics is often necessary, contributing to the spread of AMR/AMR genes (4). However, effective management and control require a comprehensive approach. Early disease diagnosis, preventive practices such as vaccinations and monitoring are crucial for mitigating the impact of respiratory diseases. Therefore, the present Research Topic aims to provide evidence to various aspects of respiratory diseases. This includes investigating immunity, exploring effective detection, comparing pathogenetic agents and examining the prevalence of respiratory diseases.

Bovine respiratory disease (BRD) has been widely studied and the most common viruses and bacteria have been identified. These include *Pasteurella multocida, Mycoplasma bovis, Histophilus somni*, and *Mannheimia haemolytica* which are closely associated with BRD. This is further confirmed by the findings of Hirsch et al., that investigated the potential association between bacterial bronchopneumonia (BP) in cattle and their procurement through auction markets. More specifically the study examined the prevalence, antimicrobial susceptibility, and genetic relatedness of these bacterial pathogens in two groups of cattle, those transported to auction markets before feedlot placement and those directly sent to feedlots from farms. Key findings revealed that transportation and co-mingling at auction markets did not significantly impact the acquisition of major respiratory pathogens, with *P. multocida* identified as the predominant pathogen. High genetic diversity was observed initially while horizontal transmission of resistant bacteria could occur in the feedlot, possibly influenced by antimicrobial administration.

BRD is a multifactorial disease influenced by various stressors, including transportation stress. Therefore, among farm management practices, preconditioning (PC) of animals shows profitability toward controlling BRD, including weaning strategies, vaccination against pathogens, to better distribute stress over time, decrease economic losses and reduce morbidity and mortality. Lower morbidity and mortality were confirmed through an interesting study of Mijar et al., where auction-derived (AD) and PC beef calves were commingled. AD animals resulted having higher morbidity and therefore increased administration of antibiotics, although no bacterial resistance was observed. Even though mortality rates were low, only AD animals died. These results are further confirmed from another study, when compared with PC animals (5). Another aspect of that study was the average daily gain (ADG) that resulted higher in AD respect to PC animals, although the emphasis was on exploring the effects of comingling.

The abovementioned studies provided important results regarding BRD. However, there is currently no gold standard for the diagnosis of BRD ante mortem. Additionally, diagnosing BRD cases is challenging due to the variability in clinical signs (6). Some of the techniques include thoracic auscultation, which might lack in sensitivity and specificity (7). The study of Donlon et al. aimed to explore the prevalence of BRD using a hierarchical Bayesian latent class model and two common yet imperfect techniques: thoracic ultrasound (TUS) and clinical respiratory scoring (CRS). The prevalence estimates were sensitive to variations in the priors for test characteristics and the case definition for thoracic ultrasound, with different thresholds resulting in different prevalence estimates. The study identified that a substantial proportion of calves with pulmonary lesions likely went undiagnosed if only examined for clinical signs using the respiratory scoring system. The analysis revealed that the prevalence of calf respiratory disease, while low, is highly variable in seasonal calving dairy herds.

Prior studies have mainly focused on the identification of viral and bacterial causes of BRD. Interestingly the comparative study by Centeno-Martinez et al., aimed to explore the fungal community in nasal cavity of both visually healthy and BRD affected animals as well as to determine bacterial-fungal co-occurrences in both test groups. Despite the absence of evidence for fungal pathogens, the findings identified *Trichosporon* and *Issatchenkia* as the most abundant genera in the nasal cavity regardless of health status. Notably, *Candida* spp. has been linked with several diseases in cattle while *Candida krusei* had been further associated with bronchopneumonia (8). The study (Centeno-Martinez et al.) also highlighted that the fungal community structure was affected by seasonal variations while cross-kingdom interactions between bacteria and fungi resulted more prevalent in BRD animals than healthy animals. However, only a few cross-kingdom interactions have been identified highlighting the complexity of microbiome and its potential role in respiratory diseases.

Transitioning from cattle to swine, pseudorabies (PR), also known as Aujeszky's disease is a viral infection primarily affecting swine caused by *suid herpesvirus* (SHV-1). Despite vaccination with the Bartha-K 61 attenuated live vaccine, PRV outbreaks still occur due to the emergence of PRV variants with stronger virulence. The study of Chang et al. aimed to develop a more efficient single dose vaccine using Astragalus saponins (AST). AST has been traditionally used in Chinese medicine known for its anti-inflammatory and antiviral properties. To evaluate the vaccine's efficacy, a mice model was utilized, demonstrating that AST increased the survival rate, especially after a lethal dose challenge with PRV. Although swine is the main host of PRV, a wide range of other hosts includes cattle, sheep, mice etc. In the case of AST-adjuvanted vaccine group, the production of gB and neutralizing antibodies was significantly enhanced. Notably, the AST-adjuvanted vaccine showed lower viral loads in the brain and lungs concluding that AST adjuvant is effective and can enhance immunity even with single dose against PRV.

Accurate and rapid diagnosis of SHV-1 is a prerequisite for effective prevention and control of PR. Current techniques include molecular diagnosis, serological testing and viral isolation and identification. Although quantitative PCR (qPCR) can accurately identify viral infections in both early and late stages, it cannot determine dead and live viruses. Therefore, the use of propidium monoazide (PMA) together with qPCR was utilized in the study of Yang et al., determining the infectivity of SHV-1. PMA, a photosensitive dye, can bind to both dead or live viruses enabling the rapid determination between infectious and non-infectious viruses. The researchers optimized the PMA-qPCR method, by selecting suitable primers, determining the optimal PMA concentration, and investigating the effects of UV or heat inactivation of the virus. UV inactivation, however, did not compromise the virus's capsid, preventing PMA from binding to its nucleic acid, rendering UV inactivation unsuitable for determining infectivity. Nevertheless, the combination of PMA and qPCR demonstrated high specificity and sensitivity in the identification of SHV-1.

The experimental prospective and comparative studies published in the present Research Topic provide an overview of the challenges for the most prevalent respiratory diseases in the livestock industry. The complexity of interactions between host animals has increased the need for accurate disease management. Despite extensive research, we still lack optimized diagnostic methods and rapid identification of the pathogen. However, these works raise questions to be further addressed in future studies.

## Author contributions

AP: Writing—review & editing. FC: Writing—review & editing. MM: Writing—original draft. BP: Conceptualization, Supervision, Writing—original draft, Writing—review & editing.

## References

[1] Blakebrough-Hall C McMeniman JP González LA. An evaluation of the economic effects of bovine respiratory disease on animal performance, carcass traits, and economic outcomes in feedlot cattle defined using four BRD diagnosis methods. J Anim Sci. (2020) 98:1–11. 10.1093/JAS/SKAA005 31930299 PMC6996507

[2] Blasi B Sipos W Knecht C Dürlinger S Ma L Cissé OH. *Pneumocystis* spp. in pigs: a longitudinal quantitative study and co-infection assessment in Austrian farms. J Fungi. (2022) 8:10043. 10.3390/jof8010043 35049984 PMC8779942

[3] Gaudino M Nagamine B Ducatez MF Meyer G. Understanding the mechanisms of viral and bacterial coinfections in bovine respiratory disease: a comprehensive literature review of experimental evidence. Vet Res. (2022) 53:70. 10.1186/s13567-022-01086-1 36068558 PMC9449274

[4] Zalewska M Błazejewska A Czapko A Popowska M. Antibiotics and antibiotic resistance genes in animal manure – consequences of its application in agriculture. Front Microbiol. (2021) 12:610656. 10.3389/FMICB.2021.610656/BIBTEX 33854486 PMC8039466

[5] Roeber DL Speer NC Gentry JG Tatum JD Smith CD Whittier JC. Feeder cattle health management: effects on morbidity rates, feedlot performance, carcass characteristics, and beef palatability. Prof Anim Scientist. (2001) 17:39–44. 10.15232/S1080-7446(15)31594-1

[6] Timsit E Dendukuri N Schiller I Buczinski S. Diagnostic accuracy of clinical illness for bovine respiratory disease (BRD) diagnosis in beef cattle placed in feedlots: a systematic literature review and hierarchical Bayesian latent-class meta-analysis. Prev Vet Med. (2016) 135:67–73. 10.1016/J.PREVETMED.2016.11.006 27931931

[7] Buczinski S Forté G Francoz D Bélanger AM. Comparison of thoracic auscultation, clinical score, and ultrasonography as indicators of bovine respiratory disease in preweaned dairy calves. J Vet Intern Med. (2014) 28:234–42. 10.1111/JVIM.12251 24236441 PMC4895545

[8] Kano R Konishi K Nakata K Sano K Komatsu S Nomura M. Isolation of *Candida krusei* from a case of bovine bronchopneumonia in a one-year-old heifer. Vet Record. (2001) 148:636. 10.1136/VR.148.20.636 11394804

